# The Factors Associated With Disease Mismanagement in Young Patients with Type 1 Diabetes: A Qualitative Study

**Published:** 2015-04

**Authors:** Selda Celik, Meral Kelleci, Ilhan Satman

**Affiliations:** 1 Department of Internal Medicine, Istanbul University, Division of Endocrinology and Metabolism, Istanbul, Turkey;; 2 Department of Nursing, Faculty of Health Sciences, Cumhuriyet University, Sivas, Turkey;; 3 Department of Internal Medicine, Division of Endocrinology and Metabolism, Istanbul University, Istanbul, Turkey

**Keywords:** Type 1 Diabetes Mellitus, Disease Management, Qualitative Research, Young Adult

## Abstract

**Background:**

The objective of this qualitative study on young adults with type 1 diabetes was to determine the factors associated with mismanagement of diabetes.

**Methods:**

In this qualitative study, a descriptive phenomenological and psychological method was followed. Purposeful sampling method was used in this study. 28 young adults aged 18-25 with type 1 diabetes (16 females, 12 males) with HbA_1_c levels >6.5% were interviewed in-depth. Each interview lasted 40-45 minutes. The recorded interviews were transcribed verbatim, examined line-by-line and coded using open coding techniques and managed by QSR NVivo 7. During the research period, Guba ve Lincolln criteria were used to ensure the accuracy and precision of the study findings.

**Results:**

The study identified seven themes which affect the diabetes management of the patients. These themes were negative emotions about the disease, difficulties arising from living condition, difficulties arising from the treatment treatment process, lack of social support, not solution oriented coping methods, concerns about the future and issues of developing knowledge and attitude regarding diabetes management.

**Conclusion:**

There are multiple factors affecting the management of diabetes in young adults with type 1 diabetes. Diabetes has a biopsychosocial impact on young adults’ lives, developing a negative attitude toward their future and that of their family.

## Introduction

Type 1 diabetes is a genetic-based condition resulting from absolute insulin deficiency due to destruction of the beta cells of the pancreas.^1^ With rapidly increasing incidence worldwide, type 1 diabetes also imposes high costs on the society. Type 1 diabetes accounts for approximately 5-10% of all diabetes cases with an estimated 78.000 new cases diagnosed annually.^2^

The main purpose of diabetes care and treatment is to maintain insulin activity and blood glucose within normal limits, thereby reducing vascular and neuropathic complications. For this purpose, a treatment plan is made in order to manage the normal daily life, diet and activities of the patient and to prevent problems due to blood glucose fluctuations. Low hemoglobin A1c (HbA1c) levels lower the risk of diabetes complications.^3^ The Diabetes Control and Complications Trial (DCCT) demonstrated that strict glycemic control significantly decreases the risk of long-term diabetes complications. However, 81-87% of patients have HbA1c >7.0%, 74% of whom have HbA1c >7.5%.^3^^,^^4^

Patients with type 1 diabetes must first of all be able to self-administer their insulin, plan their meals and a proper diet, maintain blood glucose control, exercise regularly in order to reach targeted blood glucose levels, achieve short- and long-term well being, and elevate their quality of life.^5^^,^^6^ In order to perform all of these, in other words, in order to manage diabetes, people need to change their lifestyle.^7^^,^^8^ Some studies demonstrated compliance to diet in 39%, and regular exercise only in 19%.^4^^,^^9^ For many years, non-compliance with the treatment regimen in chronic diseases has been known as 50%.^10^^,^^11^ However, compliance is a more serious problem in patients with diabetes. As mentioned above, one of the reasons is the necessity for the patients to change their lifestyles in order to manage diabetes. In addition, the multi-dimensional treatment plan for diabetes also makes compliance difficult for diabetes patients. This may also contribute to compliance problems experienced by many patients. One study has shown that patients’ complied more to drug therapy than to changing their lifestyles.^12^ A study by Salomon et al. has shown that having insufficient information about their disease and being surrounded by friends and family members with health concerns have negative effects on young adults’ perception of the disease.^13^ Another study by Serlachius et al. revealed that insufficient information and ability specific to diabetes management, and problems in finding a balance among health concerns, daily life, and self-care have an impact on their management.^14^

Young adulthood is a period which involves preparing for a future, and developmental tasks such as building a career, marriage and parenthood. The patients try to meet their developmental tasks while having concerns about the disease, life and death. Therefore, determining the perception of disease, and concerns about diet, insulin use, exercise, and future are important for the patients’ diabetes management. However, the interaction of these factors, the big picture and causes of the problem, has remained unclear. Nonetheless, it is known that accommodation of the patients to treatment processes and their behaviors of disease management are complex. But it is considered that it will be useful to use a qualitative approach in understanding these complex behaviors and determin the problems in mismanagement of the disease. 

## Materials and Method

The objective of this qualitative study on young adults with type 1 diabetes was to determine the factors associated with mismanagement of diabetes. Our aim was to contribute to improvement of new approaches in management of the disease by determining how they manage diabetes, how they perceive the disease, their experiences in the process of using diet, exercise and insulin and going for checkups. 

The study was conducted at Istanbul University, Faculty of Medicine, Department of Internal Medicine, Endocrinology and Metabolism Diseases, Diabetes Clinic. The study used a purposeful sampling method which is the most common type of qualitative sampling techniques.^15^ The aim of using this method was to recruit a sample of approximately 28 young adults with type 1 diabetes, at ages 18-25, and HbA_1_c level over 6.5%. The patients who agreed to participate in the study were included in the sampling.

The interviews were conducted by the clinic’s diabetes training nurse and the researcher who is the principal author. Each patient was interviewed once. Each interview lasted 40-45 minutes. The interviews were recorded by a recording machine upon written consent of the patients. A total of 28 young adults (16 females, 12 males) were interviewed in-depth. The demographic and background characteristics of the patients are presented in Table 1. The patients were asked the following questions during interviews: (1)What does diabetes mean to you?, (2) What should the attitude towards diabetes management be?, (3) What difficulties do you have in diabetes management?,(4) How has diabetes affected your life?, (5) How do you use insulin?, (6) Do you check your blood sugar level?, (7) Can you exercise regularly?, (8) What difficulties do you have about your diet?, (9) How is your social life affected by your disease?, and (10) Would you like to share your concerns about the future?

**Table 1:** Characteristics of the patients

**Description**	**N (%)**
Gender	
Female	16 (57.1)
Male	12 (42.9)
Age (years)*	22.2±1.9
Educational level	
Junior high school and high school	4 (14.3)
University	24 (85.7)
Relationship	
Married	8 (28.6)
Single	20 (71.4)
Duration of diabetes (years)*	8.5±5.8
HbA1c (%)*	8.5±2.1

*Data are given as ‘mean±SD’

*Ethical Considerations*

The study obtained the approval of Istanbul University, Ethics Committee. Information was provided to the interviewees about consent, confidentiality, and how the data would be used, both during recruitment and at the interview. All the participants signed a written consent form at the beginning of the interview.

*Data Analysis*

In this study, diabetes management of young adults who have type 1 diabetes and cannot keep their diabetes under control was investigated. The general structure of the case studied was based on transcripts from 28 individuals according to the recommendations of the descriptive phenomenological psychological method.^16^ Giorgi reported that such analysis included minimum five basic steps: (a) collection of verbal data, (b) reading of the data, (c) division of data into parts, (d) organization of data from a disciplinary perspective and (e) synthesis of the data. The first stage of the analytic process was preparation of an interview guide including broad and open-ended questions which enabled the participants to express their viewpoint comprehensively. After that, a word-by-word transcription was made based on the information obtained from the interviews. The topics of importance for diabetes management were transformed into questions. The interviews recorded by researchers were reviewed. After the questions’ validation was established, ambulatory patients admitted to the diabetes research and screening center were interviewed using the semi-structured interview form. Of the patients, those who were approved for the study after they were obtained from the daily list of clinic outpatient admissions and reviewed were informed of the study. In the second stage, all the transcripts were read twice to provide a sense of integrity and understand the meaning of the experiences from the participants’ viewpoint; at this stage, the researchers independently determined the themes. In the third stage, the principal author divided the transcribed data material into what Giorgi calls “meaning units” which were then confirmed by the other authors. The purpose was to separate the material into smaller parts by identifying the themes that described how the patients handled diabetes in everyday life. We ended up with a series of meaning units that were still expressed in the participants’ own words. Each meaning unit was labeled with a code representing its content. Then all the units were organized into theoretical categories, using a standard word-processing program. In the final stage, the themes identified from the interview material were synthesized.

**Figure 1:**
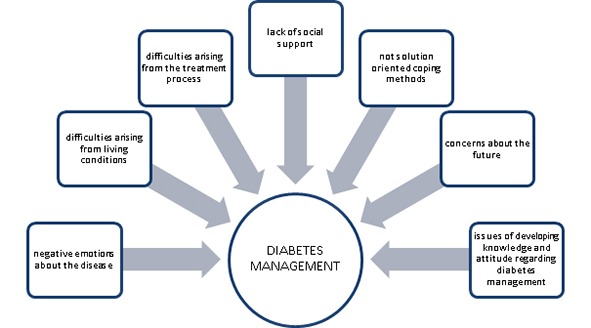
Model: the factors affecting diabetes management of young adults with type 1 diabetes.

Records were deciphered by two researchers. The recorded interviews were transcribed verbatim, examined line-by-line and coded using open coding techniques and managed by QSR NVivo 7.^17^ Categories were grouped into themes, checked by other researchers and final categories and themes negotiated (Figure 1).

*Study Rigor*

In this study, we utilized the assessment criteria of Guba and Lincoln. In the study, an atmosphere where patients can comfortably tell about themselves and answer the questions was formed. To make sure that the data is accurate and reliable, the authors deciphered the records independently. Then the deciphering was gathered together and their conformity was assessed. Data coding and theme forming processes were performed rigorously. Transferability was considered by having a team (including authors and coauthors). The final categories were determined by the entire study team. The long term experience of one of the researchers (the third author) as a counselor in substance withdrawal centers was useful in confirming the codes and interpretations. During sampling, maximum variation was considered to ensure credibility. To ensure conformability, the researches deciphered the data individually. A collective decision was finally made. The participants were informed of the answers they gave after the interview. 

## Results

The individuals who participated in the study stated that diabetes has caused significant changes in their lives and that it was difficult to adapt themselves to these changes. Themes resulted from the data analysis of the attitudes of young adults with type 1 diabetes towards disease management.

*Theme 1: Negative Emotions about the Disease*

The study revealed that young adults mostly have negative emotions during the period of acceptance of the disease. These negative emotions are fear, outrage, revolt, weariness, guilt, frustration, vagueness, despair and shame, respectively. All these emotions were causing a tendency to hide the disease and thereby stop using insulin and complying with the diet. On one hand, there were disease specific rules that had to be followed and on the other hand, relationships with friends. Consequently, these two situations were causing conflicts and eventually negative emotions. For example, one of the participants stated:

*“You’ve got to be careful all the time. This is very exhausting. I feel aggrieved. I am not like others, I am different. My friends can eat anything they like. I want to be free to eat whatever I want, too; but I can’t. I feel guilty after eating. I secretly feel anger towards my friends. And then I think, they don’t even know that I’m ill”. (F, age 21).*

It is worth noting that 25 of the young adults had negative emotional burdens about following the diet and administering insulin to maintain glycemic control which is crucial for managing the disease. *“Following an order, being limited, and the necessity to be careful about certain things all the time make me feel restricted (F, age 21)”.* Feeling restricted and the thought of having to do the same things all the time were affecting diabetes management.

In addition, these individuals stated that they felt aggrieved, they didn’t have any ill people around and they also wanted to be like normal people. The majority of them had the tendency to hide their disease. Twenty-three of those interviewed confessed that they wanted to be like normal people. 

*“My social life has changed, for example, when we go out with friends. I can’t eat anything I want, while the others can. I can’t inject insulin in front of other people. I don’t want anybody to know that I am ill. I feel humiliated when they say “Oh, what a pity”. I don’t want their mercy. Just one colleague knows about my disease. And I told her only because I may need help in case of an emergency” (F, age 22).*

*“I don’t let anyone know that I am ill. No one knows. I don’t want them to see me as a sick person” (M, age 23).*

*“ I am happily engaged. He doesn’t know that I have this disease. He thinks that I am a normal person. When I am with him, I do whatever he does, I don’t restrict myself about food, and I don’t inject insulin. Other times, I inject insulin into my leg or belly to keep him from noticing. I’m afraid of losing him.” (F, age 23).*

Eighteen patients experienced severe anger during the period of acceptance of the disease and had difficulty in anger management. 

*“I was psychologically affected, I became pessimistic. I became an angry person. I can’t even tolerate the smallest things, I get angry easily. Most of the time I say I wish I wasn’t ill” (F, age 22).*

*Theme 2: Difficulties Arising from Living Conditions*

The individuals talked about the difficulties they had faced in their professional and education life, and problems with diet and using insulin in these circumstances. 

“ *Life outside does not let me follow the necessities of my disease. I have a class, I am in the classroom. You can’t say, “I have to inject insulin or eat something”. You start life defeated 1 – 0. You worry about being pitied by others or considered different if you tell them the truth” (F, age 20).*

There were conflicts about diabetes management and responsibilities. They were having problems about regulating their life conditions. They had a problem with the realization that they have to achieve this by themselves. Irritability and blaming others were other important issues. 

*“You need to eat something right after the injection. But when you are at school, you are a student and you can’t do that during class by saying “injection time”, you can’t eat. Teachers reacted to that in the class. One time, the teacher said “The medication for diabetes is expensive, couldn’t you afford it?” He said that in class and I was quite upset. Most people don’t have any knowledge about diabetes. When my blood sugar drops, I need to eat or drink something. But my friends don’t understand this” (F, age 19).*

There were concerns about life, disease and adaptation. There was a high tendency to see the disease as an obstacle, a condition that cannot be accepted.

*“ At work, no one knows about my disease; I didn’t tell anybody. Nobody wants to work with someone who is ill. I don’t want to lose my job. I need to eat snacks between meals but there is a camera across the room. The manager monitors all the time, you can’t eat. I will lose my job if they learn that I am ill” (F, age 23).*

*“ I eat out with my friends. They can eat whatever they like. I have to accommodate myself to them. I behave like them; I can’t behave in a different way” (F, age 22).*

Two interviewees said that the fact that missing an insulin injection does not cause any symptoms affects diabetes management. They said:

*“Everything goes wrong while working; I work surrounded by people all the time. I can’t say, “I’m ill, wait a minute I’ll inject insulin, I have to eat snacks”. Naturally, the system gets disrupted. When one thing gets out of order, the others follow. Eventually, I say “Never mind, what can I do?”. When I don’t inject insulin, I don’t feel any pain or distress. I might inject if I had some distress, but it doesn’t make any difference” (M, age 22).*

Another important point is that they did not lead a healthy lifestyle before the disease. For individuals who ate a high-carbohydrate diet instead of a balanced diet, skipped meals, didn’t exercise regularly and were not living with people who did, it becomes hard to push themselves to eat 6 times a day, adopt an adequate and balanced diet and exercise after the disease. 

*“I am not fond of eating. This didn’t change when I was diagnosed either. I have never exercised. It is really hard to adopt things at a late stage” (M, age 21).*

*Theme 3: Difficulties Arising from the Treatment Process*

Some patients mentioned the difficulties arising from the attitude of the treatment team. Especially, accusatory, judgmental attitudes and lack of empathy have made it difficult for them to come for follow-ups and follow the recommendations. The fact that the suggestions were not appropriate for the patient’s life and they remained as a theory was another factor affecting their adaptation. One of those statements:

*“The doctors’ suggestions differ from one other. They say, “Eat this, don’t eat this”. But the outside environment is not always suitable for that. Their suggestions do not always fit in with social life.” (M, age 22).*

*“I am afraid to ask my doctor questions. I know that my blood sugar is high. I don’t come for check-ups for fear of being reproache” (F, age 21).*

The patients wished to be recognized by the treatment personnel. Being cared for by different health personnel each time was another factor affecting their adaptation.

*“I am not taken care of by the same person each time. Newcomers need to know me from the beginning. This is really tiresome” (F, age 21).*

Difficulty accepting the disease is reflected as a lack of tolerance to the questions asked by others. One of them stated: 

*“I wait for a long time when I come for check-ups. During that time, other people are waiting there ask questions. Do you have diabetes as well? What a shame. I thought only elderly people had this disease. They say these kinds of things. I don’t want to give any explanations; I get angry. This is why I don’t want to come for check-ups, I postpone them” (F, age 23).*

Most of the patients stated that they had trouble reaching the team and they wait too long in the queue for an examination; therefore, they don’t want to come for check-ups. 

*Theme 4: Lack of Social Support*

Patients stated that they had issues trusting their families and friends. They had worries about being deserted, pushed away by their friends and left alone. 

*“I think they will leave me when they learn that I am ill. I have no friends to stand by me. I’m trying to live like them, too” (F, age 21).*

Four of the participants stated that they felt the peer pressure of their friends, they did not know what actually their disease was and they understimated their condition. And although they don’t agree with their friends, sometimes they go along with it not to hurt their friends and feel like one of them. They had different issues with their family. One of the participants said:

*“My father does not want me to inject insulin. He wants me to use alternative medicine. He comes up with something new every day and asks me to try it. We argue about this a lot. Sometimes I might have to do what he says even if I don’t want to” (F, age 22).*

They also had to deal with protective parents and friends who try to restrict nearly everything. Patients stated that they usually dislike such attitudes, get angry and are sometimes unkind.

*“My mother tries to control everything about me. I’m very distressed” (F, age 23).*

Some of the participants said that society was prejudiced because of insufficient knowledge about the disease and this attitude made it hard for them to accept diabetes. Most of them had the tendency to think that the society feels sorry for people with diabetes. 

*“I have family problems. They don’t support me at all, they don’t care about me and they have no interest or knowledge about my disease” (M, age 23).*

*“(crying) At school, my friends’ attitudes have changed. My mother used to prepare a lunchbox for me and my friends were making fun of me saying, “Did you bring food again?” when they saw it. I got upset and felt left out. I used to go home, eat and go to bed. I wasn’t talking with anyone except my mother. “She is ill,” they would say when talking about me. Being excluded by my friends like this made me very upset, in fact I am no different from them” (F, age 18).*

*Theme 5: Not solution Oriented Coping Methods*

There were statements of feeling weak, running away from problems and blaming others. For example: 

*“I have too many problems that I need to solve. But I am not strong enough to cope with them. I ignore them” (F, age 20).*

Several participants had secondary gain from their disease and were engaged in risky behaviors.

*“I am ill and this might be an opportunity for me. I don’t place any restrictions on myself. If I want to buy a car, I somehow buy it. I take a bank loan” (M, age 22).*

*Theme 6: Concerns about the Future*

Twenty-four participants had fears and concerns about becoming permanently disabled, losing their organs and dying at an early age. 

*“My kidneys may stop functioning, my legs may be amputated. I don’t inject insulin regularly, I don’t eat healthfully, I don’t check my blood sugar at all. And then I am afraid of losing my legs. I live in fear all the time. Even so, I do nothing” (F, age 21).*

*“I am anxious about having a disabled child when I get married, or that my child will also become ill like me and have to do injections every day. That’s why I can’t cope in relationships with the opposite sex” (M, age 22).*

*Theme 7: Issues of Developing Knowledge and Attitude Regarding Diabetes Management*

All of the interviewees stated that they were aware of the necessity of taking insulin injections, following the diet and checking their blood sugar regularly for diabetes management. 24 participants said that they didn’t exercise and some of them stated that they didn’t know how to count carbohydrates. Some difficulties were reported as to managing their lifestyle and leading an orderly life.

*Injecting insulin: *Interviewees admitted that they had difficulties using insulin although they knew that they had to inject insulin in the stated dose before meals. Having to take injections at the same time every day and eat after injections was especially stressful. There is a tendency to skip doses or postpone injections. What is important here is that although they say, “I know, I am dependent on insulin, I must take it”, they haven’t adopted this attitude. One participant:

*“I get up around 10-11 in the morning, I can’t wake up early. When I take my injection late in the morning, I postpone the noon injection. I take my noon injection at 17:00. Sometimes I skip a dose. I know I shouldn’t do this but it is not intentional. I just can’t, and I can’t wake up in the morning if I sleep at 1 or 2 am. My insulin times were arranged according to my lifestyle but I couldn’t follow it, either. I don’t like being bound to things. There are things ahead of me and I have to conform to them, I can’t get used to it, I never could. I want things to be in order but I can’t do anything about it” (M, age 22).*

The participants who tended to hide the disease avoided injecting insulin in social environments; they felt uncomfortable with people staring and asking questions. In addition, they were fed up with insulin due to reasons including having to repeatedly do the same things over and over and seeing no change in their life. 

*“During the injection, inserting the needle into the skin is really hard. My legs are too rigid, the injection site hurts more, gets bruised and bleeds. It is very painful even after I take the needle out. I can’t inject in my arms, I don’t want others to see them” (F, age 21).*

*Blood sugar check: *A remarkable number of participants stated that they don’t check their blood sugar levels. Some of them said that they feel the fluctuation in blood sugar and that’s why they don’t need to check it; and others said they don’t check it because they are afraid to see the results.

*“I check my blood sugar level once a day. I am supposed to check it 3 times a day, but I don’t check it if I feel alright. I don’t want to routinely check it all the time. Sometimes seeing the result makes me upset. The most difficult one is the night check and I don’t do that one either” (M, age 22).*

*Diet:* The biggest problem was diet. They stated that they knew what not to eat, and that they had to eat three meals and three snacks. However, there were participants who didn’t know about carbohydrate counting. In addition, patients who hide their disease were not following the diet so as not to be different from others in social settings. On the other hand, the majority of participants can’t put their daily lives in order, skip meals, and don’t eat the necessary snacks. There were patients who thought having to follow things all the time was constraining and they didn’t feel free; there were also patients who overate due to misinformation and anxiety.

*“The dietician says, “Don’t eat this, be careful about that”. I don’t know about carbohydrate counting; neither have I asked nor been taught. That’s why I find it hard to regulate my diet. It’s not easy to change at once. Things get out of order more easily when I am out. I have to eat convenience foods, I become disorganized. I am supposed to eat vegetables and whole wheat, but I can’t eat those. I don’t like them. I skip snacks, I know it is necessary but I can’t follow it. I forget to, or am not hungry at all, I can’t eat much, and then I feel fatigued and light-headed. Sometimes my blood sugar drops despite the snacks. I was more careful in the past. One gets fed up over time and begins skipping more often” (M, age 22).*

*Exercising: *Exercising couldn’t be established within the lifestyle. These individuals said that they were not exercising prior to the disease. Consequently, although it has been a long time since diagnosis, they couldn’t manage to exercise due to not believing the necessity and a failure to develop a sense of control over their lives.

*“I don’t exercise; it is not a part of my lifestyle. I don’t believe that it is necessary. That’s why it has never been a habit for me; exercise doesn’t fit into my lifestyle” (F, age 21).*

On the other hand, two participants said that they knew exercising was essential but they didn’t do it due to lack of time. One of the participants said that she was living in a small town, people were very conservative, they would gossip about her if they saw her going for a walk and therefore she couldn’t exercise. One participant said that he exercised, not because he is diabetic or exercising is important in diabetes management, but because it is a part of his education. 

## Discussion

In this study, we analyzed factors which affect the young adults with type 1 diabetes in mismanagement of the disease. We examined how the patients performed insulin injections, meal planning and appropriate diet, maintaining self-check blood glucose monitoring, and exercising regularly which are important in diabetes management. We found that patients had negative feelings about their disease and had difficulties arising from living conditions, difficulties arising from the treatment process, lack of social support, were coping methods not solution-oriented, and had concerns about the future and issues of developing knowledge and attitude regarding diabetes management. These factors were associated with mismanagement of the disease by the patients. 

Our findings have shown that although the average disease duration was 8.4 years, they still had problems with accepting the disease and with psychosocial adaptation. As it is already known, disease is an existential crisis affecting the individual with its psychosocial aspects. The individual, who is stuck between life and the disease phenomenon, experiences conflicts with himself, his world and his future.^18, 19^ This emotion, thought and behavior pattern might be the reflection of personality-related problems emerging as a result of failure to reliably resolve the critical situations while growing up. On the other hand, it might be the sign of psychological problems, such as depression.^15^^,^^19^^,^^20^

We have determined 7 themes in our study. These findings will help us understand the problematic attitudes of young adults with type 1 diabetes regarding diabetes management. The study by Spencer, Cooper and Milton (2013) investigated the experiences of young adults with type 1 diabetes by means of adapting to the diagnosis, learning to live with diabetes and dependence. In the same study, the difficulties in diabetes management stated by the participants were similar to the ones in our study. The results of the study showed that providing information about diabetes management to the patients was not enough in itself; determining negative emotions, engaging social support and giving them strength to cope with the idea of accepting the disease, making it a lifestyle and learning to live with the disease must be considered. Some studies emphasized the need for social and professional support in order to achieve optimal success in diabetes management.^21^^-^^23^

In the present study, not all the patients could proceed to the acceptance stage even though the disease durations were long enough. The emotions of fear, anxiety, anger and weariness were common. The reason for these emotions was perceived as the disease itself and other people. These negative emotions were important factors affecting their ability to adapt to and accept their disease.^24^^,^^25^ Some studies suggested that negative emotions and concerns about diabetes are important factors affecting adaptation to the disease. Therefore, this negative point of view prevents young adults from disease management while indicating the risk of mood disorders. In actuality, depression is an important problem very common in these patients.^18^^,^^20^^,^^24^ The negative emotions and ideas experienced and lack of coping methods not designed for problem solution in addition to insufficient social support may lead to negative perceptions of events in the internal and external world and a feeling of being powerless to make a change. On the other hand, the problems originating from the health system in evaluating and addressing the biopsychosocial aspects may result in the condition becoming chronic. Consequently, they may get fed up with the treatment and feel exhausted. Some studies reported outcomes in favor of our study results.^26^^,^^27^

Drawing on the work of Arnett,^28^ diabetes researchers argue that young adulthood is divided into two stages. The first one is the early transitional phase (approximately 18-22 years of age) and the second one (approximately 23-30 years of age) is characterized with increased lifestyle stability.^29^^-^^31^ Young adults in their twenties have bigger future anxiety and pay more attention to their diabetes management than do their younger counterparts.^32^ They tend to lose their adolescent sense of invulnerability.^33^ As to the themes obtained from the study, particularly in depth interview forms of having concern about the future and coping with problems can be associated with these features of the young adults.

Another theme obtained in the study was the lack of social support. Being knowledgeable about how social support operates seems to be essential to improve diabetes patients’ self-care, ensure their compliance with professional advice, encourage them to adapt to new lifestyles, and help them improve outcomes of diabetes care and increase their personal freedom. The role of social support was studied in terms of several other medical conditions and healthcare in general.^34^^-^^36^

A study showed that the knowledge of the patients about their disease was incomplete and incorrect. Lack of knowledge is also one of the significant factors affecting management of diabetes. In this study, it was determined that patients with diabetes lacked knowledge about the following issues: insulin dosage adjustment during illness, suitability of food choices, frequency of need for ophthalmology review and foot care.^37^

In this study, factors related to incorrect management of diabetes by young adults with type 1 diabetes are studied. From this aspect, this study can guide clinicians, in particular diabetes nurses, in handling, understanding patients biopsychosocially and developing programs which will enable them manage diabetes effectively. Nonetheless, this study had some limitations. The sample size was large enough. This study can be repeated with a larger sample size. In addition, interviews were made with the group that came to the clinic to act as control. Considering that there can be patients who cannot manage their diabetes and appear for controls, conducting this study in the society can enable the authorities get different information. We aimed to create an overall framework in our study. The data obtained from this study is planned to be used in a training program which helps patients handle disease management more effectively. 

## Conclusion

In this study, we determined the factors affecting disease mismanagement for young adults with type 1 diabetes. The study’s primary outcomes were that negative emotions and ideas, method of coping, social support, living conditions, treatment process, concerns about the future, and problems developing knowledge and attitude towards diabetes management were all affecting disease management. In this study, we particularly showed that it is not sufficient for the healthcare team to only explain the treatment process and tell what to do in order to help the patients manage diabetes and the individual’s negative emotions and thoughts, coping skills, social support, living style and concerns about future are also effective. The number of interviews can be increased and each aspect can be individually analyzed in subsequent studies. Participants can be re-interviewed and their opinions about the themes can be received after the themes formed. 
